# Intraoperative biopsy during dacryocystorhinostomy in a patient with lacrimal sac MALT lymphoma mimicking dacryocystitis: A case report 

**DOI:** 10.3892/mi.2026.309

**Published:** 2026-03-11

**Authors:** Tatsuya Yunoki, Hirohiko Tachino, Yuka Morita, Noriko Okuno, Atsushi Hayashi

**Affiliations:** 1 Department of Ophthalmology, Graduate School of Medicine and Pharmaceutical Sciences, University of Toyama, Toyama 930-0194, Japan; 2 Department of Otolaryngology, Head and Neck Surgery, Faculty of Medicine, Academic Assembly, University of Toyama, Toyama 930-0194, Japan; 3 Department of Pathology, Academic Assembly Faculty of Medicine, University of Toyama, Toyama 930-0194, Japan

**Keywords:** lacrimal sac lymphoma, mucosa-associated lymphoid tissue lymphoma, dacryocystorhinostomy, intraoperative biopsy

## Abstract

Lacrimal sac primary tumors are uncommon and often mimic inflammatory conditions, rendering preoperative diagnosis difficult. The present study describes the case of an 83-year-old female patient presenting with left-sided epiphora and mucopurulent discharge. Based on the clinical findings, dacryocystitis was strongly suspected. Computed tomography dacryocystography revealed only a mild filling defect, with no radiological evidence suggestive of a neoplastic lesion. However, during endoscopic dacryocystorhinostomy, a well-defined polypoid lesion was observed within the lacrimal sac. A lesion biopsy revealed a mucosa-associated lymphoid tissue lymphoma. No metastasis was observed in other organs, and the patient has been under observation for 24 months post-operatively without recurrence. The present case report highlights the importance of obtaining an adequate tissue biopsy for definitive diagnosis when any mass-like or elevated lesion is encountered within the lacrimal sac, even in the absence of suspicious pre-operative findings. The intraoperative careful inspection of the entire lacrimal sac is essential in order to avoid missing malignant tumors and to enable early diagnosis.

## Introduction

Although primary tumors of the lacrimal sac are rare, they can occasionally present as a cause of nasolacrimal duct obstruction (1,2). When the tumor is confined within the lacrimal sac without extra-saccular extension, preoperative diagnosis is often challenging. Computed tomography (CT) and CT dacryocystography (CT-DCG) can improve the detection rate of space-occupying lesions within the lacrimal sac (3-5); however, small intramucosal or submucosal tumors may remain undetected on imaging. Extranodal marginal zone B-cell lymphoma of mucosa-associated lymphoid tissue (MALT lymphoma) most commonly arises in the stomach, salivary glands and ocular adnexa; however, recent studies have demonstrated that it may also occur in a variety of non-classical extranodal sites, frequently presenting with non-specific inflammatory features and leading to diagnostic delay (6-8).

Among primary lacrimal sac tumors, lymphomas are rare, and reports of MALT lymphoma are particularly uncommon (9-12). In numerous cases, these tumors are misdiagnosed as chronic dacryocystitis and are managed conservatively for extended periods of time, rendering pre-operative diagnosis especially difficult. The present study describes the case of a patient with lacrimal sac MALT lymphoma which was diagnosed intraoperatively by biopsy; although the patient exhibited typical symptoms of dacryocystitis, there were no obvious pre-operative imaging findings suggestive of a tumor.

## Case report

**Figure 1:**
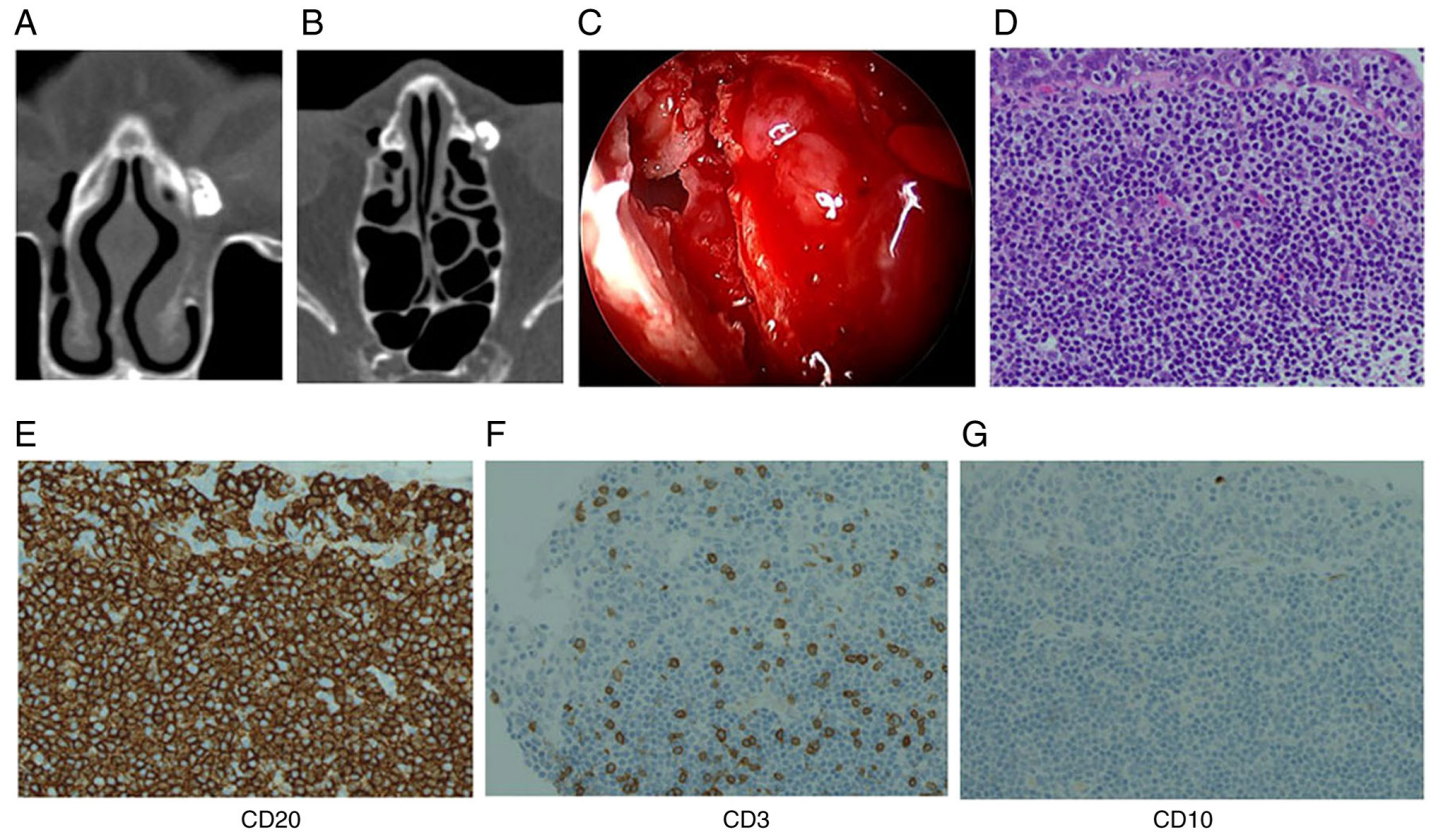
A case of MALT lymphoma within the left lacrimal sac in an 83-year-old woman. Computed tomography-dacryocystography of the left lacrimal sac: (A) Coronal plane image illustrating the lacrimal sac filled with contrast, with a mild filling defect noted. (B) Axial plane image illustrating the lacrimal sac with the same mild filling defect. Neither image indicates findings suggestive of an orbital or paranasal sinus tumor. (C) Nasal endoscopy indicating an elevated mass in the lacrimal sac. (D-G) Pathology of a surgically removed mass in the lacrimal sac. (D) Hematoxylin and eosin staining illustrating the proliferation of small atypical lymphocytes (original magnification, x400). (E) Immunostaining is diffusely positive for CD20 (original magnification, x400). (F) Scattered CD3-positive reactive T-cells are present in the background (original magnification, x400). (G) CD10 staining is almost negative (original magnification, x400). These findings support the diagnosis of MALT lymphoma. MALT, mucosa-associated lymphoid tissue.

An 83-year-old woman presented to Toyama University Hospital (Toyama, Japan) in July, 2022 with complaints of left-sided epiphora and mucopurulent discharge. Upon a pre-operative physical examination, no palpable mass was detected in the medial canthus, and gentle pressure over the lacrimal sac elicited mucopurulent reflux. A pre-operative laboratory evaluation consisted of routine blood tests, which revealed no clinically significant abnormalities other than poorly controlled diabetes mellitus (HbA1c, 8.1%). She had a medical history of diabetes mellitus, but no history of immunosuppressive therapy or any malignancy. No bloody discharge was observed. Lacrimal irrigation revealed the complete obstruction on the left side with purulent reflux. Representative images of CT-DCG are illustrated in Fig. 1. A coronal plane image is presented in Fig. 1A and an axial plane image is illustrated in Fig. 1B, both demonstrating the lacrimal sac filled with contrast, with a mild filling defect noted. A mild filling defect was noted, which was not definitively suggestive of a neoplasm. Endoscopic endonasal dacryocystorhinostomy (DCR) was performed. Intraoperatively, a well-circumscribed polypoid mucosal mass was identified within the lacrimal sac, and a biopsy was obtained (Fig. 1C).

A histopathological examination was performed using 4-µm-thick sections obtained from formalin-fixed, paraffin-embedded tissue. The specimens were fixed in 10% neutral-buffered formalin at room temperature for ~24 h. Hematoxylin and eosin staining was performed according to standard protocols. Staining was carried out at room temperature. The stained sections were examined using a standard light microscope routinely used in the pathology laboratory (manufacturer not recorded). The histopathological examination revealed a uniform proliferation of small lymphocytes infiltrating the epithelium (Fig. 1D). In addition, immunohistochemical analysis was performed on 4-µm-thick sections prepared from formalin-fixed, paraffin-embedded tissue. Following deparaffinization and antigen retrieval using standard protocols, the sections were incubated with primary antibodies against CD20, CD10, and CD3. The antibodies used were commercially available antibodies purchased from Roche Diagnostics (Ventana Medical Systems): CD20 (clone L26, cat. no. 760-2531), CD10 (clone SP67, cat. no. 790-4506), and CD3 (clone 2GV6, cat. no. 790-4341). These antibodies are routinely used in th authors' pathology laboratory. Immunostaining was carried out according to standard protocols at room temperature, followed by visualization using a conventional detection system routinely used in our pathology laboratory; however, the supplier and catalogue number of the detection system were not recorded at the time of analysis A commercially available secondary antibody routinely used in our pathology laboratory was applied; however, the dilution, catalogue number, supplier, conjugate, and the exact temperature and duration of incubation were not recorded at the time of analysis. Sections were counterstained with hematoxylin according to standard protocols at room temperature. Immunostained sections were examined using a light microscope (manufacturer not recorded). The magnification is indicated in the figure legends. Immunohistochemical staining revealed positive reactivity for CD20, almost negative for CD10, and scattered CD3-positive reactive T-cells in the background (Fig. 1E-G), leading to a diagnosis of MALT lymphoma. In the differential diagnosis, IgG4-related disease, reactive lymphoid hyperplasia and other types of malignant lymphoma were carefully considered. IgG4-related disease was considered unlikely as there were no histopathological features such as dense storiform fibrosis or obliterative phlebitis, and the patient had no systemic manifestations suggestive of IgG4-related disease. In addition, there was no histopathological evidence suggestive of IgG4-related disease, and serum IgG4 levels were within the normal range. Reactive lymphoid hyperplasia was excluded based on the presence of a monotonous proliferation of small lymphoid cells with lymphoepithelial lesions and the lack of preserved follicular architecture. Other B-cell lymphomas, including follicular lymphoma and diffuse large B-cell lymphoma, were considered unlikely due to the absence of a follicular growth pattern, only a weak and focal expression of CD10, the lack of high-grade cytological atypia and low-grade histological features.

Taken together, these morphological and immunohistochemical findings supported the diagnosis of MALT lymphoma. Genetic testing and flow cytometry were not performed. Comprehensive clinical staging was performed to determine whether the lesion represented primary or secondary involvement. Whole-body positron emission tomography-computed tomography (PET-CT) was performed for staging purposes and revealed no evidence of systemic lymphoma involvement, and a bone marrow examination revealed no evidence of lymphomatous infiltration. Based on these staging results, the lesion was diagnosed as a primary lacrimal sac MALT lymphoma. The patient has been under careful observation without additional treatment. Follow-up has primarily been performed with periodic CT scans, and no recurrence has been observed during 24 months of follow-up.

## Discussion

In the present case report, the patient exhibited typical clinical features of dacryocystitis, and CT-DCG revealed only a mild filling defect within the lacrimal sac, with no findings suggestive of malignancy. However, the intraoperative identification of a polypoid lesion prompted a biopsy, which revealed MALT lymphoma. Primary malignant lymphoma of the lacrimal sac is rare and often clinically indistinguishable from inflammatory conditions. MALT lymphoma is known to arise in a wide range of extranodal sites beyond the classic locations and often presents with non-specific inflammatory features, which can result in diagnostic delay. The present case report reflects this well-recognized diagnostic challenge, with lacrimal sac involvement mimicking chronic dacryocystitis (6-8). Accordingly, differentiating primary lacrimal sac MALT lymphoma from chronic dacryocystitis can be particularly challenging due to overlapping clinical features.

Suspicious findings that may suggest a lacrimal sac tumor include a firm medial canthal mass, recurrent dacryocystitis, bony erosion on imagingand blood-stained epiphora (13). However, these signs are typically absent in the early stages of disease. Notably, blood-stained epiphora is uncommon and has been reported to be a non-specific and infrequent symptom in patients with lacrimal sac malignancies (13). As demonstrated by the present case report, some tumors may present without any characteristic symptoms and are diagnosed incidentally during surgery. Previous pathological studies analyzing routinely excised lacrimal sac tissue during DCR have reported that the majority of cases exhibit non-specific inflammation, with the incidence of malignant neoplasms ranging from 0 to 3.7% (14-18). Based on these findings, several studies have suggested that routine lacrimal sac biopsy during DCR may be unnecessary and should be reserved for cases with intraoperative findings suspicious for neoplasm (14-18). However, the available evidence in the literature remains limited regarding which specific intraoperative features should prompt a tissue biopsy.

Previous studies have indicated that 22-38% of patients with malignant lacrimal sac tumors initially present with symptoms resembling dacryocystitis (1-3). Moreover, it has been reported that 60-88% of lacrimal sac tumors are malignant in nature (12,19). Among these malignancies, squamous cell carcinoma and malignant lymphoma are the most common histological types (12,20). Given these findings, it is essential not to overlook the possibility of malignancy, even in cases without pre-operative radiologic or clinical suspicion. In the event that any mass-like or elevated lesion is observed within the lacrimal sac during surgery, a proactive and definitive biopsy should be performed. The key lesson from the case described herein is the potential diagnostic pitfall in lacrimal sac lesions presenting with symptoms indistinguishable from chronic dacryocystitis. Even when pre-operative imaging findings are non-specific, small intramucosal or submucosal lymphomas may be concealed within the lacrimal sac. Careful intraoperative inspection of the entire lacrimal sac is therefore crucial, and any mass-like or elevated lesion should prompt biopsy to avoid delayed or missed diagnosis.

Where possible, additional diagnostic modalities, such as flow cytometry and genetic analysis are recommended to ensure accurate characterization of the lesion. In the case presented herein, clonality testing was not performed, which represents a limitation of the present case report. Nevertheless, the diagnosis was supported by characteristic histopathological and immunohistochemical findings. This underscores the importance of careful and thorough intraoperative inspection of the entire lacrimal sac to avoid missing potentially malignant lesions and facilitate early diagnosis and appropriate management.

The management of ocular adnexal MALT lymphoma varies depending on disease stage and institutional practice, and treatment options may include observation, radiotherapy, or systemic therapy (6,10).

Based on these findings, even in the absence of preoperative signs suggestive of a tumor, any mass-like or elevated lesion identified within the lacrimal sac should be biopsied with adequate tissue sampling to obtain a definitive diagnosis. In the present case report, the patient has been followed-up primarily with periodic CT scans, and no recurrence was observed during 24 months of follow-up. While magnetic resonance imaging may provide higher soft-tissue contrast and may be considered if clinical suspicion arises or if CT findings are inconclusive, CT was deemed sufficient for routine monitoring given the location and size of the lesion.

The present case report highlights the importance of maintaining a high index of suspicion for neoplastic disease during surgical treatment of lacrimal sac disorders. It serves as a valuable reminder of the clinical significance of thorough intraoperative evaluation and reinforces the need for vigilance in the diagnosis and management of lacrimal sac tumors.

## Data Availability

The data generated in the present study are not publicly available in order to protect patient privacy but may be requested from the corresponding author.
